# Emergence of Mixed Mode Oscillations in Random Networks of Diverse Excitable Neurons: The Role of Neighbors and Electrical Coupling

**DOI:** 10.3389/fncom.2020.00049

**Published:** 2020-06-08

**Authors:** Subrata Ghosh, Argha Mondal, Peng Ji, Arindam Mishra, Syamal K. Dana, Chris G. Antonopoulos, Chittaranjan Hens

**Affiliations:** ^1^Physics and Applied Mathematics Unit, Indian Statistical Institute, Kolkata, India; ^2^The Institute of Science and Technology for Brain-Inspired Intelligence, Fudan University, Shanghai, China; ^3^Department of Mathematics, Centre for Mathematical Biology and Ecology, Jadavpur University, Kolkata, India; ^4^Division of Dynamics, Faculty of Mechanical Engineering, Lodz University of Technology, Lodz, Poland; ^5^Department of Mathematical Sciences, University of Essex, Colchester, United Kingdom

**Keywords:** Izhikevich neuron model, random networks, bicurcation scenaria, mixed mode oscillations (MMOs), mixed mode bursting oscillations (MMBOs), excitable neurons, electrical coupling

## Abstract

In this paper, we focus on the emergence of diverse neuronal oscillations arising in a mixed population of neurons with different excitability properties. These properties produce mixed mode oscillations (MMOs) characterized by the combination of large amplitudes and alternate subthreshold or small amplitude oscillations. Considering the biophysically plausible, Izhikevich neuron model, we demonstrate that various MMOs, including MMBOs (mixed mode bursting oscillations) and synchronized tonic spiking appear in a randomly connected network of neurons, where a fraction of them is in a quiescent (silent) state and the rest in self-oscillatory (firing) states. We show that MMOs and other patterns of neural activity depend on the number of oscillatory neighbors of quiescent nodes and on electrical coupling strengths. Our results are verified by constructing a reduced-order network model and supported by systematic bifurcation diagrams as well as for a small-world network. Our results suggest that, for weak couplings, MMOs appear due to the de-synchronization of a large number of quiescent neurons in the networks. The quiescent neurons together with the firing neurons produce high frequency oscillations and bursting activity. The overarching goal is to uncover a favorable network architecture and suitable parameter spaces where Izhikevich model neurons generate diverse responses ranging from MMOs to tonic spiking.

## 1. Introduction

Diverse spiking oscillations and bursting phenomena of electrical activity in single neurons or neuronal networks play an important role in information processing and transmission across different brain areas (Connors and Gutnick, 1990; Izhikevich, 2003, 2004, 2007; Coombes and Bressloff, 2005; Antonopoulos et al., 2015, 2019; Ma and Tang, 2017; Mondal and Upadhyay, 2018; Teka et al., 2018). The underlying mechanism of signal processing in neurons depends on the variations of membrane voltages called spikes (Izhikevich, 2003, 2004, 2007). The complexity of spikes or trains of spikes can be controlled by external stimuli, e.g., by injected electrical currents. In a common scenario, a bunch of spikes (called a burst) may emerge in the activity of single neurons or in neural populations (Izhikevich, 2000; Coombes and Bressloff, 2005; Constantinou et al., 2016; Zeldenrust et al., 2018). Such oscillatory patterns of membrane voltages can be modeled mathematically by biophysical dynamics (with realistic parameters) such as the (un)coupled Izikevich neuron model (Khoshkhou and Montakhab, 2018), described in the next section. Our goal is to study the firing and collective activities of coupled neurons in an environment of heterogeneous excitabilities. Neural networks support functional mechanisms within brain areas. For example, such diverse groups of neurons in the cortex are responsible for many complex neuronal mechanisms (Izhikevich, 2000, 2004, 2007).

Most of the neurons are excitable, i.e., they show quiescent behavior however, they can also fire spikes when they are stimulated by input stimuli. In neural computations, the neurons continue to fire a train of spikes when there is an input by injecting a pulse of direct current (DC) and this is called tonic spiking. There exist different types of spiking patterns depending on the nature of the intrinsic dynamics. Bursting follows a dynamic state in a neuron where it repeatedly fires discrete groups or bursts of spikes, i.e., when the activity alternates between a quiescent state and repetitive spiking (a bunch of spikes appear together). This might be regular or chaotic, depending on the dynamics of the system and excitabilities or couplings (Izhikevich, 2000, 2004, 2007). Apart from spiking and bursting activities, one of the interesting complex firing patterns emerge from the activity of neurons is the mixed-mode oscillations (MMOs) (Brøns et al., 2008; Desroches et al., 2012; Bacak et al., 2016), what is the main focus here. In MMOs, the oscillations are distributed with different amplitudes where the firings alternate between large and small amplitude oscillations (Brøns et al., 2008) (i.e., the so called *LAO*s and *SAO*s, respectively) reflecting different rhythmic activities such as locomotion or breathing (Bacak et al., 2016). The multiple time scales (e.g., fast potassium channels with slow kinetics; Ghaffari et al., 2015) of voltage variables or controlled noise can induce MMOs in neuronal systems (Muratov and Vanden-Eijnden, 2008; Upadhyay et al., 2017). MMOs were first observed in chemical reaction systems (Ostwald, 1900). They were also observed in Belouzov-Zhabotinsky reactions (Schmitz et al., 1977; Showalter et al., 1978; Brøns and Bar-Eli, 1991), calcium dynamics and electrocardiac systems (Kummer et al., 2000; Rotstein and Kuske, 2006). We note that, from a dynamical perspective, the generation of MMOs can be analyzed through the canard phenomenon (Eckhaus, 1983; Drover et al., 2004; Rubin and Wechselberger, 2008) and also via homoclinic bifurcations (Chakraborty and Dana, 2010). Krupa et al. (2008) analyzed the mechanism of MMOs in a two-compartmental model of dopaminergic neurons in the mammalian brain stem. To investigate the generation of MMOs in a self-coupled, FitzHugh-Nagumo model, Desroches et al. (2008) developed a computational method and Guckenheimer (2008) examined how chaotic dynamics and MMOs arise near folded nodes and folded saddle-nodes on slow manifolds. Vo et al. (2010) demonstrated that MMOs can generate a type of bursting that can be reflected in a biophysical model of pituitary lactotroph (Toporikova et al., 2008). MMOs were also observed in stellate cells of the medial entorhinal cortex (layer II) and Rotstein et al. (2008) analyzed the mechanism of such patterns in a biophysical, conductance-based, model. Apart from MMOs, mixed-mode bursting oscillations (MMBOs) (Desroches et al., 2013) were also observed when a bunch of spikes in a single burst appears with *SAO*s. In MMBOs, burst activity appears instead of single spikes within LAOs. Our study on network dynamics sheds more light on such interesting patterns.

In this paper, we explore the emergence of spiking and MMOs in a random network of diffusively coupled (through the membrane voltage variable) Izhikevich neurons in a backdrop of diverse excitabilities. The role of network structure and arrangement of mixed neural populations in the network are the main objectives for the study of the emergence of MMOs. In network neuroscience, researchers investigate the firing activities and collective patterns of neural activity where neurons are connected in a complex-network topology (Brøns et al., 2008; Desroches et al., 2008; Erchova and McGonigle, 2008; Postnov et al., 2008; Krupa et al., 2014; Malagarriga et al., 2015; Antonopoulos, 2016; Borges et al., 2017, 2020; Khoshkhou and Montakhab, 2018). For instance, a correlated synchronous firing appears in neuronal cells with the adaptive exponential integrate-and-fire model with excitatory-inhibitory synapses that can be associated with epileptic seizures (Protachevicz et al., 2019). Bittner et al. (2017) showed that balanced excitatory and inhibitory input currents in clustered (non-clustered) networks of neurons may reflect spiking activities in which inhibitory neurons share more coherent activities. Recently, MMOs have also been observed in pre-Bötzinger complex networks (Bacak et al., 2016) (a medullary region that controls breathing in mammals) in the presence of heterogeneous excitable parameters. In both studies, a three-coupled reduced model was proposed to understand the behavior of collective spiking patterns and the conditions for the emergence of *LAO*s and *SAO*s were studied.

However, the role of network architecture and different excitabilities in the emergence of MMOs are not well-understood. In this paper, we have affirmative answer to the question related to the emergence of MMOs. We reveal how such MMOs can be distinguished from other firing patterns, supported by their relevant biophysical significance (Golomb, 2014). Moreover, the neurons in the paper are placed on the nodes of a random network and transfer signals through its links. In the absence of coupling, the activity of the considered neuronal population reveals two types of dynamical states (or excitabilities), ranging from spike-bursting to subthreshold to quiescent states. The key question that arises here is the following: considering a mixed/heterogeneous neural population (neighboring neurons of self-sustained spiking neurons might have subthreshold oscillations), can we design a random network of neurons (with Poissonian neighbor node-degree-distribution) that will give rise to collective firings where subthreshold or quiescent neurons are compelled to show high amplitude activities? We want to uncover the coupling parameter space and the ratio of mixed populations where MMOs and fast tonic spiking behavior emerge. In this context, by mixed/heterogeneous neural population we mean that neurons with different excitability properties i.e., the non-identical neurons with different firing patterns are connected in a complex network. At weak couplings and a diluted random network setting, we show that desynchronized subthreshold neurons exhibit MMOs. With the increase of the coupling, all subthreshold neurons fire in a mixed-mode state. In both cases, MMOs are not prominent in oscillatory neurons and eventually disappear as the coupling strength increases. Consequently, neural subpopulations emerge as synchronous clusters exhibiting tonic spiking behavior. For diluted random and homogeneous networks, where the electrical coupling strength is constant, we show that neighbors exhibiting self-sustained oscillations, determine the structural patterns of MMOs. Based on the synchronized cluster over a certain coupling range, we can reduce the random network to a low dimensional, reduced-order network, i.e., to two coupled oscillators which reflect and predict the diverse dynamical patterns that appear in the random network. Additional to the random network, we have validated our results in small-world network of 500 nodes. In particular, our results for both types of networks confirm that the emerging features observed in the random network can also be found in the small-world network.

The paper is organized as follows: in section 2, we describe the Izhikevich neuron model and discuss its dynamical properties. The model displays various electrical activities (i.e., different spiking and bursting patterns) for fixed parameter values and for a range of injected currents, *I*. Then, we investigate the dynamical behavior on a random network (see section 2.2) based on single Izhikevich neurons with various firing responses. In particular, we identify the parameter region and coupling strategy where MMOs and MMBOs exist, and analyze the transition phases of firing responses (sections 2.2.1 and 2.2.2). In section 3, the reduced-order network model is constructed to verify the results obtained for the random network. A bifurcation analysis is also performed to show the mixed mode states and other phases of oscillations. In section 4, the MMOs are further tested in a small-world network. Finally, we conclude our work in section 5, followed by a discussion.

## 2. Biophysical Model and Random Network

### 2.1. Model Description

Our work focuses on the analysis of the complex dynamical behavior in the 2-dimensional nonlinear Izhikevich model that captures neuronal membrane voltages (Izhikevich, 2003, 2004). It produces spiking and bursting patterns distributed over a range of parameter values. It is a biophysically plausible and computationally efficient mathematical model that takes into account continuous spike generation and a discontinuous resetting process following the spikes. It has two state variables; the membrane voltage, *v* and recovery variable, *u*, which measure the activation of K^+^ and inactivation of Na^+^ ionic currents, respectively. The dynamical activity of an Izhikevich neuron is captured by the set of equations

(1) $$\begin{array}{l} \overset{∙}{v}=0.04v^{2}+5v+140-u+I, \end{array}$$

(2) $$\begin{array}{l} \overset{∙}{u}=a\left(bv-u\right), \end{array}$$

with an after-spike resetting constraint, i.e., when the membrane voltage *v* reaches a peak value *v*_*pk*_, the following relation is applied: if *v* ≥ *v*_*pk*_(= 30), then *v* ← *c* and *u* ← *u* + *d*. The parameters *a*, *b*, *c*, and *d* are dimensionless. The resting potential ranges in the interval −70 to −60mV and depends on *b* that indicates the sensitivity of *u* to the subthreshold fluctuations of the membrane potential, *v*. The parameter *a* measures the timescale of the recovery variable *u*. The parameters *c* and *d* control the after-spike reset value of *v* and *u*, respectively, caused by fast high-threshold K^+^ channel conductances and slow Na^+^ and K^+^ conductances. The function (0.04*v*^2^ + 5*v* + 140) was derived using the spike initiation dynamics of a cortical neuron. The different suitable choices of parameters generate various types of oscillations, often found in neocortical and thalamic neurons (Connors and Gutnick, 1990; Gray and McCormick, 1996; Izhikevich, 2000). The initial conditions are set to *v* = −63 and *u* = *bv*. Synaptic currents or injected DC-currents are delivered via *I*. We consider a fixed parameter regime that produces different firings for a single Izhikevich neuron (Izhikevich, 2003, 2004), i.e., *a* = 0.1, *b* = 0.2 with reset parameters *c* = −65 and *d* = 8, what we call set I. We note that for *I* < 4, the system of Eqs. (1) and (2) does not show any spiking or bursting behavior. Thus, the firing patterns can be obtained for *I* ≥ 4. Simulations of the systems of ordinary differential equations were performed using the fourth-order Runge-Kutta method with a fixed time step of 0.01, as the simulation results with a smaller time step did not show any significant differences. Bifurcation diagrams of the deterministic dynamical model in the reduced-order network were computed using the MatCont software package (Dhooge et al., 2003).

### 2.2. Formulation of the Network of Model Neurons

**Figure 1:**
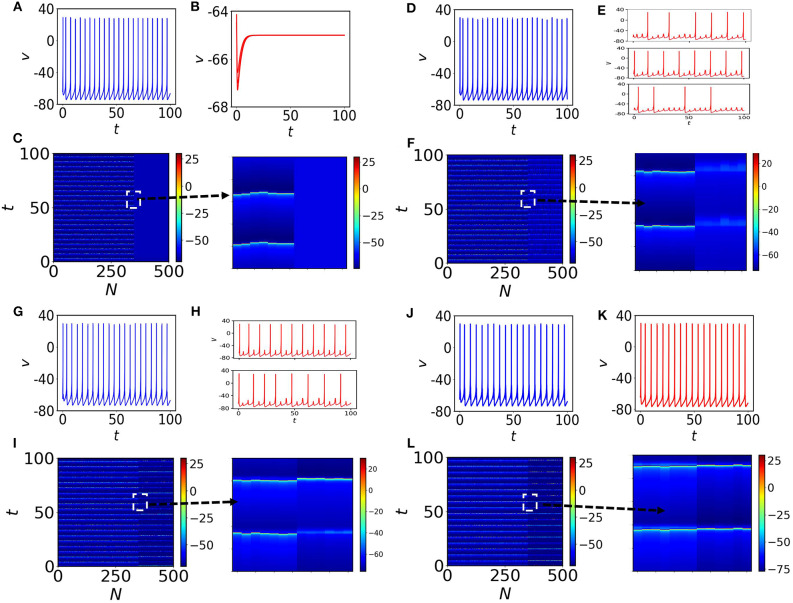
Membrane potential *v* and spatiotemporal plots. **(A)** One self-oscillatory spiking neuron in the absence of coupling (*K* = 0) and a time-series of a quiescent node is shown in **(B)**. **(C)** The spatiotemporal plot for all neurons in the random network. The first 350 nodes are self-oscillatory. Nodes from 351 to 500 are in steady states (see the 4 zoom-ins). **(D,E)** The coupling is increased to *K* = 0.3. There are several types of MMOs observed in the quiescent subpopulation. Three nodes from the quiescent subpopulation are marked and the time series of each node over the course of time is shown in **(E)**. **(F)** Spatiotemporal plot of all neurons in the random network. The quiescent nodes are desynchronized with each other. **(G,H)** The coupling is increased to *K* = 0.4. *ISI* of spiking nodes are increased and decreased for quiescent nodes. Desynchronized MMOs (shown in **(H)**, where two quiescent nodes have been randomly chosen) are still visible in the quiescent population. **(I)** Spatiotemporal plot that shows the variation in spikes for all nodes in the random network. **(J–L)** are for *K* = 1. The entire population fires (without any MMOs appearing) with almost the same frequencies. Clearly two subpopulation are separately synchronized.

We construct an Erdős-Rényi (ER) random network of *N* = 500 nodes with average node-degree 5. Then, we set up a mixed population of Izhikevich neurons to model neural activity on the nodes of the random network, where 70% of them exhibit oscillatory behavior (self-sustained spiking oscillations, for *I* = 10) as shown in Figure 1B (in blue) and 30% are in quiescent states (for *I* = 3), shown in Figure 1B (in red) by setting all the parameters in the tonic spiking condition (see set I). The system is coupled via the membrane voltage *v* with a mean-field diffusive coupling. In particular, the equations of the *N* coupled neurons (*i* = 1, 2, …, *N*) in the network are described by

$$\begin{array}{l} {\overset{∙}{v}}_{i}=0.04v_{i}^{2}+5v_{i}+140-u_{i}+I_{i}+\frac{K}{\sum_{j=1}^{N}A_{ij}}\overset{N}{\underset{j=1}{\sum }}A_{ij}\left(v_{j}-v_{i}\right),\\ {\overset{∙}{u}}_{i}=a\left(bv_{i}-u_{i}\right), \end{array}$$

with the constraint that if *v*_*i*_ ≥ 30, then, *v*_*i*_ ← *c* and *u*_*i*_ ← *u*_*i*_ + *d*. *A* is the adjacency matrix of the random network, *K* the coupling strength and $S_{i}=\overset{N}{\underset{j=1}{\sum }}A_{ij}$ the degree of the *i*th node. We consider *I*_1_ = … = *I*_*p*_ = 3, where $\frac{p}{N}=0.3$ and *I*_*p*+1_ = … = *I*_*N*_ = 10, where $q=1-\frac{p}{N}=0.7$ that lead to the time evolution shown in Figures 1A,B. In the absence of coupling, the oscillatory nodes (70%) show desynchronized spiking and the rest of them (30%) converge to fixed points (see spatiotemporal plot in Figure 1C, where the inset is a zoom-in). With the increase of the coupling strength *K*, the quiescent neural subpopulation exhibits different transitions to oscillatory behavior. Generally, for weak coupling, this subpopulation generates MMOs and subthreshold oscillations. One type of MMOs shows that between two consecutive *LAO*s, there exist two *SAO*s. Interestingly, other aperiodic MMOs may coexist in this subpopulation. Interspike intervals (*ISI*) are not identical and the number of small amplitude spikes in *SAO*s within two large amplitude spikes may vary in the entire signal. We have found three types of MMOs shown in Figure 1E, randomly picked from the quiescent subpopulation in which the average interspike intervals, 〈*ISI*〉, differ significantly. We will analyze such mixed MMOs behavior and variation of *SAO*s between *LAO*s in the next subsections. This study unveils the generation and annihilation of MMOs within a subpopulation of neurons. We note that, the oscillatory subpopulation shows almost coherent tonic spiking (Figure 1D). The spatiotemporal plot of all nodes is shown in Figure 1F, where quiescent nodes are desynchronized (a zoom-in is shown on the right). With further increase of the coupling (*K* = 0.4), the quiescent subpopulation exhibits MMOs, however the number of *LAO*s between two spikes is considerably decreased. The distance between two consecutive spikes is also decreased compared to the previous coupling case, therefore, 〈*ISI*〉 is also decreased (see Figure 1H, where two randomly chosen nodes have been depicted in the panels of the figures. Interestingly, the oscillatory subpopulation remains in the same firing regime and the network shows asynchronous behavior (Figures 1G,I) for all nodes. Finally, for *K* = 1, the complete population switches to tonic spiking (Figures 1J–L) with almost identical 〈*ISI*〉, and the two subpopulations form two clusters when they are separately synchronized.

#### 2.2.1. MMOs in the Quiescent Subpopulation: Impact of Spiking Neighbors of Quiescent Nodes

**Figure 2:**
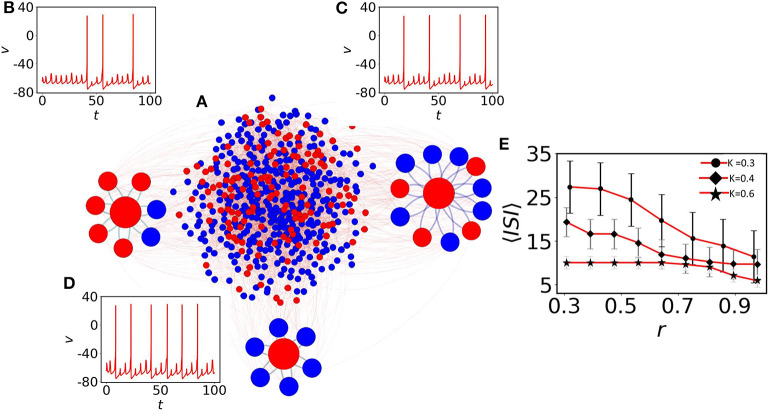
The impact of neighbors of MMOs on quiescent nodes. **(A)** The random network of 500 nodes (Bastian et al., 2009). Red nodes are in quiescent and blue in self-oscillatory states. **(B)** One red node is identified with degree 7. Five of them are spiking oscillators (*r*≈0.28). Irregular MMOs are observed here. **(C)** The second red node with *r*≈0.63. MMOs with considerably lower *ISI* are shown. **(D)** All neighbors are self-oscillatory (*r* = 1), MMOs with highly frequent spikes are observed. For **(B–D)**, the coupling strength is fixed at *K* = 0.3. **(E)** Impact of *r* on 〈*ISI*〉. The 〈*ISI*〉 is continuously decreased if we check for higher values of *r* and the average value saturates below 15 (red curve with black filled circles, red curve with black filled diamonds) for *K* = 0.3 and 0.4, respectively. For even higher coupling (*K* = 0.6, red curve with black filled stars), *r* contributes less to 〈*ISI*〉 with the value fluctuating between 5 and 10.

Here, we elaborate on the quiescent population and on several coexisting MMOs that emerge. Figure 2A shows the network structure with a mixed population (spiking neurons are shown with blue filled circles and quiescent nodes with red filled circles). We first observe the emergence of MMOs in the quiescent nodes at weak coupling. At *K* = 0.3, we have isolated three red nodes with different neighbor distributions. The red node (left) with 7 neighbors shows MMOs in which three large amplitude spikes exist within 100 time units (see Figure 2B). *ISI* are not constant and the number of small amplitude spikes between two large amplitude consecutive spikes is also varied in *SAO*s. The neighbors of this node have two silent (blue) and five oscillatory nodes (red). The number of spikes is slightly increased for another neuron originally in a quiescent state (Figure 2C) and the number of small amplitude spikes in *LAO*s is varied from 4 to 5. This neuron has 11 neighbors in which 7 nodes are self-oscillatory (blue) in the absence of coupling.

Next, we define the parameter *r*_*i*_ to search for the presence of oscillatory nodes in the neighborhood of quiescent node (*i*) by

(3) $$\begin{array}{l} r_{i}=\frac{N_{oi}}{\sum_{j=1}^{N}A_{ij}}=\frac{N_{oi}}{S_{i}}, \end{array}$$

where *N*_*oi*_ is the number of spiking oscillators connected with the *i*th quiescent node and *S*_*i*_ the degree of the *i*th node. The neighbors of a third selected node are all oscillatory (*r* = 1) and the node reveals lower *ISI* as there is comparably fast switching from *SAO*s to *LAO*s (see Figure 2D). Therefore, the ratio of adjacent spiking nodes (blue) with respect to neighbors, *S*_*i*_, determines the effect of the average *ISI*, 〈*ISI*〉, on the *i*th quiescent node (red). To understand the effect of the average *r* on 〈*ISI*〉, we have considered three couplings: *K* = 0.3, 0.4, and 0.6, shown in Figure 2E with upper red line (filled circle), middle red line (filled diamond) and lower red line (star), respectively. For the weaker couplings *K* = 0.3 and *K* = 0.4, and for small *r*, 〈*ISI*〉 exhibits significantly higher values (25 time units with high fluctuations). For higher values of *r* ≈ 1, 〈*ISI*〉 is decreased by 10 time units. The results confirm that, a red node with smaller *r* (where the presence of red (quiescent) neighbors is significantly larger, have strong impact on the red node) reduces the number of spikes compared to the case where *r* ≈ 1. For even higher couplings (*K* = 0.6, red line with star marker), 〈*ISI*〉 decreases to around 5 and the impact of *r* on〈*ISI*〉 is not prominent at even higher couplings (not shown herein). We note that, as we have seen in Figures 2B–D, smaller changes in *r* ($r=\frac{2}{7}\approx 0.28$, $r=\frac{7}{11}\approx 0.63$ and *r* = 1 for (b), (c) and (d), respectively) result in small amplitude spikes in *SAO*s between two large amplitude spikes (*LAO*s). 〈*ISI*〉 and spikes in *SAO*s of quiescent nodes are determined by two key factors: the number of neighboring spiking neurons and the coupling strength. Therefore, we conclude that 〈*ISI*〉 decreases if the number of oscillatory nodes in the neighbor increases.

#### 2.2.2. MMOs of Quiescent Nodes: The Role of Electrical Coupling

**Figure 3:**
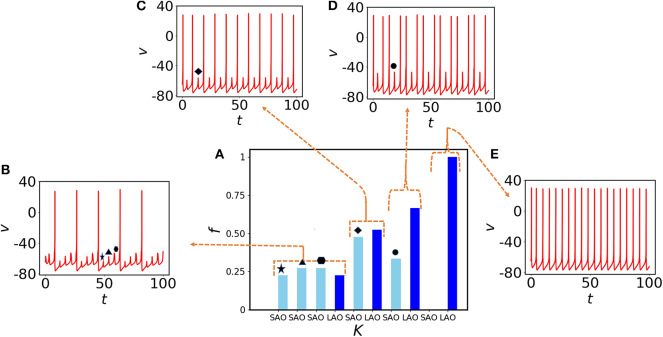
Impact of coupling *K* on MMOs of a quiescent (red) node. **(A)** Probability distribution of spikes in *SAO*s (light blue) and *LAO*s (deep blue) for *K* = 0.3, 0.4, 0.6, and 1 from left to right, respectively. **(B)** The time evolution for *K* = 0.3. Three small amplitude oscillations (star, triangle, and hexagon) appear between two consecutive large amplitude spikes. **(C)** One small amplitude spike (diamond) appears between two large amplitude spikes at *K* = 0.4. **(D)** One small amplitude spike (black circle) appears after two spikes emerging together for *K* = 0.6. Therefore, the probability of small amplitude spikes is decreased [third part of **(A)**] and results to the emergence of MMBOs. **(E)** Small spikes vanish at higher coupling (*K* = 1), therefore MMOs are lost and tonic spikes are generated, instead.

Next, we choose randomly a quiescent node (red) and check the effect of electrical coupling strength on MMOs connected to that node. At the lower coupling *K* = 0.3, the node exhibits three small amplitude spikes (*SAO*s) between two large amplitude spikes (Figure 3B). To quantify the spike distribution, we define

$$\begin{array}{l} f_{SAO}=\frac{S_{SAO}}{S_{all}},\\ f_{LAO}=\frac{S_{LAO}}{S_{all}}, \end{array}$$

where *S*_*SAO*_, *S*_*LAO*_ are the numbers of small and large amplitude spikes, respectively, and *S*_*all*_ the count of all spike amplitudes in the same interval. In Figure 3B, three small amplitude spikes appear consecutively and are shown by star, triangle, and hexagon markers, respectively. They are distributed with almost similar amplitudes (see left part of Figure 3A shown in light blue). As the membrane voltage is periodic, *f*_*LAO*_ shares almost equal probability with *f*_*SAO*_. We note that, we have used *f* in Figure 3B instead of *f*_*SAO*_ or *f*_*LAO*_ to accumulate the information of the entire spiking frequency set. If we increase the coupling to *K* = 0.4, we see that three small amplitude spikes converge to a single one (Figure 3C, diamond marker), the oscillatory neighbors influence the oscillation of the quiescent node and they are equiprobable (the light and deep blue bars in Figure 3A are almost of the same amplitudes). At *K* = 0.6, the small amplitude spikes appear recurrently (circle marker in Figure 3D) after two large amplitude spikes and give rise to MMBOs. Interestingly, simple MMOs change into more complex dynamics, i.e., MMBOs. Therefore, *f*_*LAO*_ (deep blue bar) is higher than *f*_*SAO*_ for small amplitude spikes (light blue bar). When the coupling is set to 1, the MMOs are completely lost (no light blue bar appears in the right-hand side of Figure 3A, see also the spiking behavior in Figure 3E). The quiescent neighbors at weak coupling contribute strongly to the generation of mixed-mode oscillations. When we increase the coupling, more information is shared among nearest neighbor nodes and long distant neighbors. The dynamics in the network, including that of quiescent nodes, is characterized by large amplitude spikes. We note that, the nodes in the random network are dominated by self-oscillatory neurons (70%) and for higher coupling, they control the spiking behavior in the entire network, therefore quiescent nodes cannot reflect MMOs for higher couplings.

#### 2.2.3. Average *ISI* vs. Coupling Strength *K* in Neural Subpopulations

**Figure 4:**
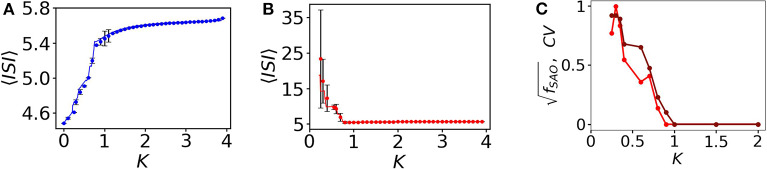
〈*ISI*〉, $\sqrt{f_{SAO}}$ and *CV* as a function of coupling *K*. **(A)** 〈*ISI*〉 for all spiking oscillators (in total 350). At small coupling, 〈*ISI*〉 is smaller, i.e., the spike frequencies are comparatively higher and it saturates around 5.6 for higher couplings. The fluctuations are negligible here, i.e., all spiking nodes have common frequencies for all couplings considered. **(B)** Quiescent nodes. For small couplings, the nodes exhibit diverse desynchronized MMOs (shown in black, with error bars). 〈*ISI*〉 saturates at higher couplings. **(C)** Relation between *CV* (red line with marker) and $\sqrt{f_{SAO}}$ (brown line with marker) as a function of the coupling strength *K*.

Here, we scan the average *ISI*, 〈*ISI*〉, interval of the entire subpopulation varying the coupling strength *K*. The 〈*ISI*〉 of oscillatory (blue) nodes in the network is slightly increased (see Figure 4A with filled blue circles) for weaker couplings and saturates around 5.6 time units when it is increased (for *K* > 1.2). On the other hand, the 〈*ISI*〉 of red quiescent nodes is decreased when the coupling is increased. For small couplings, 〈*ISI*〉 shows strong fluctuations (shown by black lines with error bars in the backdrop of red filled circles, Figure 4B) due to the desynchronized 〈*ISI*〉 in MMOs of the quiescent nodes. The red and blue lines in Figures 4A,B are plotted from the two coupled reduced models derived from the collective behavior of the connected network described in the next section. For small couplings, we see that the 〈*ISI*〉 of each quiescent node are dissimilar (see Figure 2), i.e., the firing rate varies from one node to another. We scan the entire average *ISI* interval of the quiescent subpopulation for a range of coupling strengths to understand the fluctuations in *ISI*. To quantify these fluctuations, we calculate the coefficient of variation, *CV*, of *ISI* of the quiescent subpopulation calculated from the numerical data (Figure 4C, red line with dots). *CV* becomes zero after a certain coupling strength, as there is no variation in spike sequences and *SAO*s completely vanish. The brown line in Figure 4C reflects the frequency of peaks in the *SAO*s, which is zero for higher couplings, where *CV* is also zero, thus revealing a close relation between *CV*^2^ and *f*_*SAO*_. In the Supplementary Material, we present an analytical approach that relates the two quantities and offer a plausible explanation for the discrepancy observed for small coupling strengths.

## 3. Reduced Model Description

**Figure 5:**
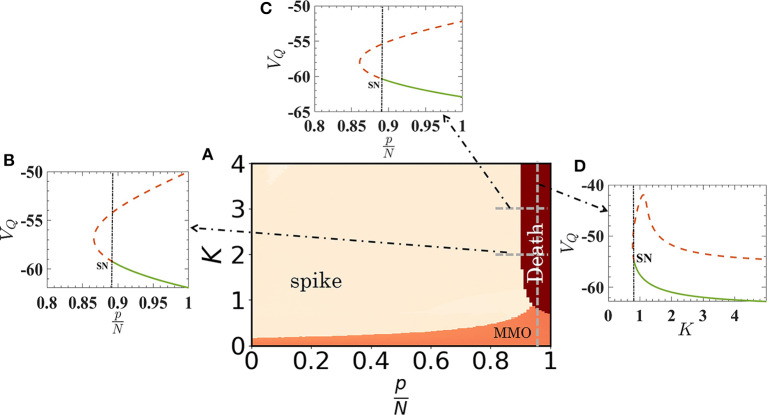
Phase-space diagram of the reduced quiescent node model as a function of *K* and relative size of quiescent oscillators in the random network. The emergence of MMOs, synchronized spiking oscillations and quiescent states are depicted in orange, pink, and dark red, respectively. The boundaries of quiescent states with other regimes are demarcated by the bifurcation scenario. **(B,C)** Stable fixed points vanish through a saddle-node (SN) bifurcation at $\frac{p}{N}\approx 0.87$ for *K* = 2 and 3, closely matched with the phase diagram. Note that for higher couplings, the boundary of quiescent states does not depend on $\frac{p}{N}$. **(D)** Bifurcation analysis as a function of *K*, for $\frac{p}{N}=0.95$ [dashed vertical line in **(A)**]. The onset of quiescent states occurs at *K* ≈ 0.77.

It is clear from Figure 1 that neurons within subpopulations are synchronized for higher couplings, and cluster synchronization appears within subpopulations. This motivates us to pursue further an approach to construct a reduced model of two coupled systems which is able to encode the information in the large network. Since we have considered a random network in which the node-degrees follow the Poisson distribution, we can approximate the degree of each node/neuron by the average degree of the considered network (Hens et al., 2015; Sasai et al., 2015). Therefore, we can assume that *S*_*j*_ = 〈*S*〉 for *j* = 1, …, *N*. The number of spiking oscillators in the neighborhood of each oscillator is expected to be $\left(1-\frac{p}{N}\right)S=\frac{q}{N}S$ and that of quiescent oscillators, $\frac{p}{N}S$, where *p* is the number of quiescent oscillators in the network. We set *v*_*j*_ = *V*_*Q*_ for *j* = 1, …, *p* and *v*_*l*_ = *V*_*S*_ for *l* = *p* + 1, …, *N*. Over a certain coupling strength, within different clusters, the quiescent and spiking oscillators are synchronized separately. Therefore, by representing the two clustered subpopulations by two nodes, we obtain the following reduced system of coupled equations

(4) $$\begin{array}{l} {\overset{∙}{V}}_{S}=0.04V_{S}^{2}+5V_{S}+140-U_{S}+I_{S}+Kp\left(V_{Q}-V_{S}\right), \end{array}$$

(5) $$\begin{array}{l} {\overset{∙}{U}}_{S}=a\left(bV_{S}-U_{S}\right), \end{array}$$

(6) $$\begin{array}{l} {\overset{∙}{V}}_{Q}=0.04V_{Q}^{2}+5V_{Q}+140-U_{Q}+I_{Q}+Kq\left(V_{S}-V_{Q}\right), \end{array}$$

(7) $$\begin{array}{l} {\overset{∙}{U}}_{Q}=a\left(bV_{Q}-U_{Q}\right), \end{array}$$

with the constraint equation that if *V*_*Q*_ ≥ 30, then *V*_*Q*_ ← *c* and *U*_*Q*_ ← *U*_*Q*_ + *d*. These conditions are also valid for spiking nodes, i.e., for Eqs. (4) and (5) for spike oscillators with *I*_*S*_ = 10 and for Eqs. (6) and (7) for quiescent oscillators with *I*_*Q*_ = 3. We note that, for homogeneous networks, there will be no effect of the assortativity (degree-degree correlation) on MMOs or on collective firing states as the number of quiescent oscillators in the neighborhood of each oscillator will not be affected. The 〈*ISI*〉 plotted for *V*_*S*_ and *V*_*Q*_ as a function of *K* is shown in Figures 4A,B with red and blue dots, respectively. The results almost match with the result for the random network (filled blue and red circles). A phase diagram of the coupled reduced model with respect to $\frac{p}{N}$ and *K* is shown in Figure 5A. The diagram is drawn by monitoring *V*_*Q*_. The MMOs and spike regions are identified with the help of *f* and quiescent (death) states by noting the variation of the peak values of *V*_*Q*_. The dark-red regime is the steady state island, where all neurons in the random network remain in quiescent states. The regime of MMOs appears for weak couplings (for all *p*) shown in orange. The uncoupled quiescent nodes are desynchronized in this regime. All nodes collectively (and individually) fire at higher couplings for *p* < 0.9 (pink region). The boundaries of each region are consistent with the results from the random network. To confirm further the onset of steady states, we have performed a bifurcation analysis to check the boundaries while we have changed $\frac{p}{N}$ from 0.8 to 1 for coupling strengths *K* = 2 and *K* = 3, respectively (see Figures 5B,C). The stable fixed point, *V*_*Q*_, is shown with thick green line in both cases. This fixed point (node) collides with a saddle point and vanishes at $\frac{p}{N}\approx 0.87$. The system shows spiking oscillations below $\frac{p}{N}\approx 0.87$ in both cases. Finally, for $\frac{p}{N}=0.95$, the system changes its dynamics from MMOs to a steady state at *K* ≈ 0.77, as evidenced in Figure 5D.

## 4. Emergence OF MMOS in a Small-World Network

**Figure 6:**
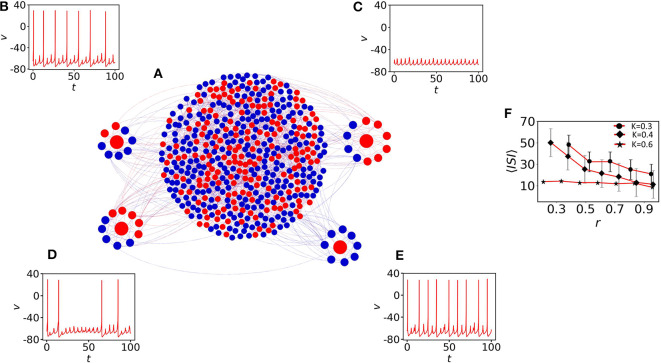
The impact of neighbors of MMOs on quiescent nodes. **(A)** The small-world network of 500 nodes (Watts and Strogatz, 1998) with *p* = 0.2 and 〈*S*〉 = 8. **(B)** One red node (quiescent) is identified with node-degree 8. Six of them are spiking oscillators (*r* = 0.75). Irregular MMOs are observed here. **(C)** The second red node with *r* ≈ 0.4. The node shows sub-threshold oscillations only. **(D)** 50% of the neighbor nodes are spiking oscillators and irregular spikes appear with high 〈*ISI*〉. **(E)** All neighbors are self-oscillatory (*r* = 1) and MMOs with highly frequent spikes are observed. For **(B–E)**, the coupling strength is fixed at *K* = 0.3. **(F)** Impact of *r* on 〈*ISI*〉. The 〈*ISI*〉 is continuously decreased if we increase *r*. The average value saturates below 30 (red curve with filled circles) for *K* = 0.3 and converges to 10 (red curve with black filled diamonds) for *K* = 0.4. *r* contributes less to 〈*ISI*〉 with the value fluctuating around 10 for *K* = 0.6 (red curve with black filled stars).

Following up the previous studies on a random network of neural computation, we construct here a small-world network of *N* = 500 nodes. A closed non-local ring is constructed with 8 adjacent neighbors. A rewire strategy (Watts and Strogatz, 1998) is implemented with a probability 0.2 to construct the final network (see Figure 6A). To understand the impact of oscillatory neighbors (i.e., blue nodes) (see Equation 3) on quiescent nodes (red), we have identified four quiescent nodes (red) with different *r*. The network comprises 40% quiescent nodes. Nodes with higher percentage of oscillatory neighbors show spiking and irregular MMOs that appear between two successive spikes (Figures 6B,E, where *r* = 0.75 and 1, respectively). However, the red nodes with a smaller percentage of oscillatory neighbors are unable to fire (*r* ≈ 0.4, Figure 6C) or irregular spikes appear with higher 〈*ISI*〉 value (*r* = 0.5, Figure 6D). The coupling strength is fixed at *K* = 0.3. Figure 6E shows the impact of *r* on 〈*ISI*〉, which is seen to continuously decrease for nodes with large percentage of oscillatory neighbors (*r* ≫ 0.1). The average 〈*ISI*〉 saturates below 30 (red curve with black filled circles) for *K* = 0.3. For this coupling strength, diverse MMOs can be seen in Figures 6B–E. For the higher coupling strength *K* = 0.4, 〈*ISI*〉 converges to 10 (red curve with black filled diamonds). *r* contributes less to 〈*ISI*〉 with the value fluctuating around 10 for *K* = 0.6 (red curve with black filled stars).

## 5. Conclusions

In this paper, we sought to study MMOs in a random and a small-world network of diverse excitable Izhikevich neurons for different coupling strengths by introducing the generation of complex oscillations. We have observed MMBOs, which are periodic in nature and are relevant to the GnRH model neuron as the dynamical behavior of these neurons in a small-size network can be useful in the studies for epilepsy (Desroches et al., 2013). We have confirmed that a certain mixed population of quiescent and oscillatory nodes can give rise to several types of MMOs and MMBOs in the two types of networks. MMOs have potential applications in biophysical and other systems. In complex systems, various mechanisms exist during different oscillatory phases that generate spike patterns between fast and slow amplitude motion together with spikes and subthreshold oscillations, termed MMOs. It was observed that pyramidal neurons are capable of exhibiting two types of MMOs and their characterization was analyzed under antiepileptic drug conditions (Babak et al., 2017). Small amplitude oscillations (<10mV) give rise to intrinsic neuronal phenomena that exist during the synaptic transmission block (Alonso and Llinás, 1989; Zemankovics et al., 2010). Actually, it has been observed in many types of neurons, such as in neurons in the thalamus, hippocampal CA1 neurons, neocortex neurons, spinal motor neurons, etc. (Puil et al., 1994; Gutfreund et al., 1995; Narayanan and Johnston, 2007; Iglesias et al., 2011). It was suggested that MMOs can be responsible for the transition from high firing rates to quiescent states by reducing neuronal gain (Iglesias et al., 2011; Golomb, 2014). Many studies showed the impacts of small amplitude oscillations/subthreshold oscillations (STOs) on diverse neuronal responses such as spike clustering (Puil et al., 1994; Gutfreund et al., 1995; Narayanan and Johnston, 2007), synaptic plasticity (Narayanan and Johnston, 2007; Bazzigaluppi et al., 2012), rhythmic activities, synchronization (Acker et al., 2003; Engel et al., 2008), etc.

Here, random networks with various injected electrical current stimuli go through different transition phases of oscillations for various coupling strengths and emerging STOs with spikes, i.e., MMOs. First, the depolarization in membrane voltages show small amplitude oscillations around steady state potentials, and with further depolarization, gives rise to spikes, e.g., to MMOs (Jalics et al., 2010). STOs play an important role in the emergence of MMOs and in controlling spike clustering (Torben-Nielsen et al., 2012; Latorre et al., 2016).

Furthermore, MMOs play an important role in neuronal functional mechanisms, namely, the STOs affect the sensitivity of neurons for injected input stimuli, the amplification of synaptic inputs and network synchronization to specific firing frequencies (Babak et al., 2017). The mechanism of MMOs produced in complex dynamical systems remains a challenging task. In the excitable pituitary cell model, pseudo-plateau bursting is canard-induced MMOs (Vo et al., 2010). It correlates electrophysiological behavior of *SAO*s on clustering spikes, and shows the influences of ionic currents to the firing rate and spike patterns in the network.

Finally, experimental and numerical studies show that MMOs occur in oscillatory rhythms in brain functioning from a single neuron to global neural networks (Erchova and McGonigle, 2008). In this study, we investigated both types of oscillations, MMOs and MMBOs. The results may be useful to Neuroscientists and those working on the mathematical modeling and dynamical behavior of cortical neurons based in random neural networks. We plan in a future publication to explore the impact of excitatory and inhibitory connections in Izhikevich neurons and how they give rise to the emergence of MMOs (Noback et al., 2005; Deco et al., 2014; Pastore et al., 2018).

## Data Availability Statement

All datasets generated for this study are included in the article/Supplementary Material.

## Author Contributions

CH and AMo designed the research study and developed the results. SG, AMo, and CH designed the figures. SG performed the analytical and numerical simulations. CH, CA, and AMo wrote the manuscript with support from SD and PJ. SD, AMi, PJ, and CA provided support by constructive suggestions and feedback.

## Conflict of Interest

The authors declare that the research was conducted in the absence of any commercial or financial relationships that could be construed as a potential conflict of interest.
