# Supplementary material for: Emergence of Mixed Mode Oscillations in Random Networks of Diverse Excitable Neurons: The Role of Neighbors and Electrical Coupling
Source: Front Comput Neurosci. 2020 Jun 8;14:49. doi: 10.3389/fncom.2020.00049 (PMC7294985; doi:10.3389/fncom.2020.00049)
Supplement: Supplementary file 1 [file Data_Sheet_1.pdf]

Emergence of mixed mode oscillations in random networks of diverse excitable neurons: the role of neighbors and electrical coupling

Supplementary Material

Subrata Ghosh[1], **Argha Mondal**[1], **Peng Ji**[5,*], **Arindam Mishra**[2], **Syamal K. Dana**[2,3], **Chris G. Antonopoulos**[4], **Chittaranjan Hens**[1,*]

[1] *Physics and Applied Mathematics Unit, Indian Statistical Institute, Kolkata, India*
[2] *Centre for Mathematical Biology and Ecology, Department of Mathematics, Jadavpur University, Kolkata, India*
[3] *Division of Dynamics, Faculty of Mechanical Engineering, Lodz University of Technology, Lodz, Poland*
[4] *Department of Mathematical Sciences, University of Essex, Wivenhoe Park, UK*
[5] *The Institute of Science and Technology for Brain-inspired Intelligence, Fudan University, Shanghai, China*

Correspondence*:
Chittaranjan Hens and Peng Ji
chittaranjanhens@gmail.com, pengji@fudan.edu.cn

1 RELATION BETWEEN CV AND F_{SAO}

To understand the relationship between the coefficient of variation, CV and f_{SAO}, we consider that the spikes in LAOs appear with probability f_{LAO} and peaks in SAOs with probability $f_{SAO} = 1 - f_{LAO}$. Furthermore, we assume that we have a sequence of spike-time intervals $\{T_{LAO}, \ldots, T_{SAO}, \ldots, T_{LAO}\}$. Based on the Bernoulli process (Golomb, 2014), if T_{LAO} appears with probability f_{LAO} in the entire sequence, then kT_{LAO} (where k is an integer with $k \geq 2$) will appear with probability $(1 - f_{LAO})^{k-1} f_{LAO}$. Therefore,

$$
\begin{aligned}
\langle ISI \rangle_n &= \sum_{k=1}^{n} kT_{LAO}(1 - f_{LAO})^{k-1} f_{LAO} \\
&= f_{LAO} \sum_{k=1}^{n} kT_{LAO}(f_{SAO})^{k-1} \\
&= f_{LAO} T_{LAO} \frac{d}{d(f_{SAO})} \sum_{k=1}^{n} \left((f_{SAO})^k \right).
\end{aligned}
$$

Setting $f_{SAO} = x \in [0, 1)$, we have that

$$\begin{aligned}\sum_{k=0}^{n} x^k &= \frac{1 - x^{n+1}}{1 - x} \\ &= \frac{1 - x^{n+1}}{1 - x} - 1 \\ &= \frac{x(1 - x^n)}{1 - x}.\end{aligned}$$

Thus,

$$\sum_{k=1}^{n} f_{SAO}^k = \frac{f_{SAO}(1 - f_{SAO}^n)}{1 - f_{SAO}}.$$

Next, we compute $\langle ISI \rangle_n$

$$\begin{aligned}\langle ISI \rangle_n &= f_{LAO} T_{LAO} \frac{d}{d(f_{SAO})} \left(\frac{f_{SAO}(1 - f_{SAO}^n)}{1 - f_{SAO}} \right) \\ &= f_{LAO} T_{LAO} \left(\frac{n(f_{SAO})^{n+1} - (n+1)(f_{SAO})^n + 1}{(1 - f_{SAO})^2} \right),\end{aligned}$$

and, in the limit of $n \to \infty$, i.e., $\lim_{n\to\infty}$, we have

$$\langle ISI \rangle = f_{LAO} T_{LAO} \frac{1}{(1 - f_{SAO})^2} = \frac{T_{LAO}}{f_{LAO}}, \tag{1}$$

where $f_{LAO} = 1 - f_{SAO}$.

Then,

$$\begin{aligned}\langle ISI^2 \rangle_n &= \sum_{k=1}^{n} k^2 T_{LAO}^2 (1 - f_{LAO})^{k-1} f_{LAO} \\ &= f_{LAO} \sum_{k=1}^{n} k^2 T_{LAO}^2 (f_{SAO})^{k-1} \\ &= f_{LAO} T_{LAO}^2 \sum_{k=1}^{n} k^2 (f_{SAO})^{k-1} \\ &= f_{LAO} T_{LAO}^2 \left(- \frac{d}{d(f_{SAO})} \sum_{k=1}^{\infty} (f_{SAO})^k + \frac{d^2}{d(f_{SAO})^2} \sum_{k=1}^{\infty} (f_{SAO})^{k+1} \right).\end{aligned} \tag{2}$$

Manipulating Eq. (2) further, in the limit of $n \to \infty$, we get that

$$\begin{aligned}\lim_{n\to\infty} \langle ISI^2 \rangle_n &= \langle ISI^2 \rangle \\ &= f_{LAO} T_{LAO}^2 \left(\frac{2}{f_{LAO}^3} - \frac{1}{f_{LAO}^2} \right).\end{aligned} \tag{3}$$

Combining Eqs. (1) and (3), we find that

$$\begin{aligned} CV &= \frac{\left(\langle ISI^2\rangle - \langle ISI\rangle^2\right)^{\frac{1}{2}}}{\langle ISI\rangle} \\ &= \frac{\left(f_{LAO}T_{LAO}^2\left(\frac{2}{f_{LAO}^3} - \frac{1}{f_{LAO}^2}\right) - f_{LAO}^2T_{LAO}^2\frac{1}{f_{LAO}^4}\right)^{1/2}}{f_{LAO}T_{LAO}\frac{1}{f_{LAO}^2}} \\ &= \frac{\left(\left(-f_{LAO}^{-1} + 2f_{LAO}^{-2}\right) - f_{LAO}^{-2}\right)^{1/2}}{f_{LAO}^{-1}} \\ &= (1 - f_{LAO})^{1/2} = (f_{SAO})^{1/2}, \end{aligned}$$

thus,

$$CV = f_{SAO}^{1/2},$$

where $CV \geq 0$ and f_{SAO} range in the interval $[0, 1)$.

To validate our theoretical analysis, we have plotted CV vs $\sqrt{f_{SAO}}$ in Fig. 1 here for a wide range of couplings K in $[0, 2]$. One can see that they follow a linear relationship. In particular, for higher coupling $K \in [1, 2]$, both CV and $\sqrt{f_{SAO}}$ tend to zero (near the origin in Fig. 1, see also Fig. 4(c) in the paper). However, for weak coupling (i.e., for K in $[0, 1]$), these quantities deviate from each other and reside away from the origin (these points are depicted in the right top corner in Fig. 1, see also Fig. 4(c) in the paper). This ensures the existence of MMOs. The discrepancy appears due to the small sample size used to compute them, as we have considered integer k values in the calculations above. In the future, we plan to explore the possibility that k assumes real values in $[0, \infty)$.

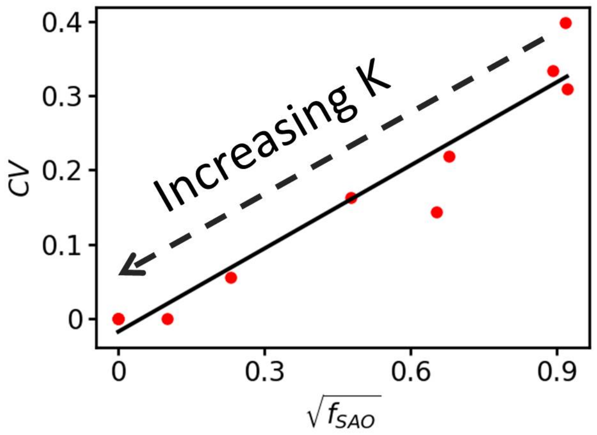

Figure 1. Linear relation between CV and $\sqrt{f_{SAO}}$. The coupling strength K is varied in $[0, 2]$ and the arrow shows the direction of increasing K in $[0, 2]$.

REFERENCES

Golomb, D. (2014). Mechanism and function of mixed-mode oscillations in vibrissa motoneurons. *PLoS One* 9, e109205
